# Population pharmacokinetics and clinical assessment of linezolid in pediatric bacterial infections

**DOI:** 10.1128/aac.01299-24

**Published:** 2025-04-01

**Authors:** Xue Tian, Tingting Jiang, Lei Dong, Xinfang Zhang, Weiwei Jiao, Gang Liu, Qinjing Li, Jing Bi, Dianping You, Ling Cao, Wenhui Guo, Zhipeng Jin, Qunqun Zhang, Yongsheng Xu, Wei Zhao, Hui Qi, Yi Zheng, Adong Shen

**Affiliations:** 1 Beijing Key Laboratory of Pediatric Respiratory Infection Diseases, Key Laboratory of Major Diseases in Children, Ministry of Education, National Clinical Research Center for Respiratory Diseases, Laboratory of Respiratory Diseases, Beijing Pediatric Research Institute, Beijing Children’s Hospital, Capital Medical University, National Center for Children’s Health 656228 , Beijing, China; 2 Hebei Key Laboratory of Infectious Diseases Pathogenesis and Precise Diagnosis and Treatment, Baoding Key Laboratory for Precision Diagnosis and Treatment of Infectious Diseases in Children, Baoding Hospital of Beijing Children's Hospital, Capital Medical University 117984 , Hebei, China; 3 Department of Pharmacy, Children’s Hospital of Hebei Province 557309 , Shijiazhuang, Hebei, China; 4 Department of Clinical Pharmacy, Institute of Clinical Pharmacology, Key Laboratory of Chemical Biology (Ministry of Education), NMPA Key Laboratory for Clinical Research and Evaluation of Innovative Drug, School of Pharmaceutical Sciences, Cheeloo College of Medicine, Shandong University 66555 , Jinan, Shandong, China; 5 Department of Infectious Diseases, National Center for Children’s Health, Key Laboratory of Major Diseases in Children, Ministry of Education, Research Unit of Critical Infection in Children, Chinese Academy of Medical Sciences, Beijing Children’s Hospital, Capital Medical University 117984 , Beijing, China; 6 Pediatric Research Institute, Children’s Hospital of Hebei Province 557309 , Shijiazhuang, Hebei, China; 7 Respiratory Department, Children’s Hospital Affiliated to Capital Institute of Pediatrics 36776 , Beijing, China; 8 Pediatric Intensive Care Unit, Children's Hospital Affiliated to Zhengzhou University, Henan Children's Hospital, Zhengzhou Children's Hospital 595848 , Zhengzhou, Henan, China; 9 Department of Respiratory, Tianjin Children’s Hospital (Children’s Hospital of Tianjin University), Tianjin, China; 10 NMPA Key Laboratory for Clinical Research and Evaluation of Innovative Drug, Qilu Hospital of Shandong University, Shandong University 91623 , Jinan, Shandong, China; University Children's Hospital Münster, Münster, Germany

**Keywords:** linezolid, children, population pharmacokinetics, efficacy and safety assessment, Monte Carlo dose simulation

## Abstract

The pharmacokinetic profile of linezolid still needs further definition, and insufficient or excessive exposure may lead to treatment failure or development of adverse events. Our study aimed to establish a population pharmacokinetic (PPK) model for linezolid in children with bacterial infections, develop an optimal dosage, and evaluate its efficacy and safety. A total of 157 plasma samples from 80 patients were utilized in PPK modeling. A one-compartment model with first-order elimination was most suitable for describing the PK characteristics of linezolid. Weight and creatinine clearance were the significant covariates for clearance. The outcomes of Monte Carlo revealed that in children under 12 years, the probability of target attainment (PTA) for standard dosage (10 mg/kg q8h) was over 90.0% when minimum inhibitory concentration (MIC) ≤2 µg/mL, with a mere 1.4% probability of surpassing the safety threshold. Meanwhile, in children aged 12 years and above, the PTA for standard dosage (600 mg q12h) was over 83.0%, and the probability of surpassing the safety threshold was 0.0%. To take the results one step further, a total of 67 patients (using standard dosage) were enrolled in the efficacy and safety analysis. Of the patients, 95.5% were cured or improved clinical treatment outcomes, and 22.4% of the patients developed possible adverse events (AEs), and no patient experienced early discontinuation of linezolid due to AEs. The standard dosage of linezolid is effective and safe in children with bacterial infections (MIC ≤2 µg/mL). For pathogens with MIC >2 µg/mL, it is advisable to switch antibiotics or increase dosage.

**CLINICAL TRIALS:**

This study is registered with Chinese Clinical Trial Registry as ChiCTR 2200061207**.**

## INTRODUCTION

Linezolid is the first oxazolidinone antibiotic that inhibits bacterial protein synthesis and prevents bacterial reproduction by binding to the bacterial 23S ribosomal RNA of the 50S subunit blocking the formation of a functional 70S initiation complex (1, 2). It is approved for treating community-acquired pneumonia, nosocomial pneumonia, bacteremia, and skin and soft tissue infections induced by Gram-positive bacteria, such as *Staphylococcus aureus*, *Streptococcus pneumoniae*, vancomycin-resistant *Enterococci*, *Streptococcus pyogenes*, and so on (1, 3–5).

In children, the regimen of 10 mg/kg, q8h, intravenous (i.v.), for children under 12 years; 600 mg, q12h, i.v., for children aged 12 years and above has been suggested for clinical treatment. Linezolid is rapidly distributed to well-perfused tissues, with a volume of distribution at steady state of approximately 40–50 L. The primary metabolism of linezolid is the oxidation of the morpholine ring, which yields two inactive ring-opening carboxylic acid metabolites, the aminoethoxyacetic acid metabolite (A), and the hydroxyethylaminoacetic acid metabolite (B). Non-renal clearance accounts for approximately 65% of the total clearance of linezolid. At steady state, approximately 30% of the drug is excreted in the urine as linezolid, 40% as metabolite B, and 10% as metabolite A. The low renal clearance of linezolid is suggestive of reabsorption into the renal tubular network. In fact, linezolid is absent from the feces, with approximately 6% and 3% of the drug appearing in the feces as metabolites B and A, respectively (1). However, studies in patients with renal failure suggest that a decrease in dose may not be necessary (3). The clearance of linezolid is a little higher, and the elimination half-life is shorter (3–4 h) in children than in adults (1).

Although several studies have established the population pharmacokinetic (PPK) model of linezolid in children and performed dose simulation, they did not evaluate the outcomes by clinical practice simultaneously. In addition, the pharmacokinetic (PK) profile and pharmacodynamic (PD) target of linezolid in children still need to be further defined. Insufficient exposure can result in therapy failure and bacterial resistance (6, 7). Conversely, excessive exposure can lead to the development of adverse events (AEs), which include gastrointestinal disturbances, confusion, hematologic toxicity, and so on (8–10).

Therefore, this study aimed to establish a PPK model for linezolid, investigate the factors affecting its PK parameters, develop a dosing regimen based on the PPK model, and evaluate the efficacy and safety of linezolid in treating bacterial infections in children using PD target based on the PPK model and real-world data.

## MATERIALS AND METHODS

### Study design

This was a prospective, multi-center study conducted in Beijing Children’s Hospital, Capital Medical University, Beijing Children’s Hospital Baoding Hospital, Capital Medical University, Hebei Children’s Hospital, Children’s Hospital of Capital Institute of Pediatrics, Henan Children’s Hospital, and Tianjin Children’s Hospital from March 2021 to June 2022. This study consisted of two parts: A total of 80 children with bacterial infections were recruited for PPK analysis, and then, to further evaluate the results of Monte Carlo dose simulation, 67 of them were subsequently included in the efficacy and safety analysis.

In the PPK analysis, the inclusion criteria were (i) children aged 0–16 years, (ii) confirmed or suspected bacterial infection (11), and (iii) treated with standard dose of linezolid for over 48 h. The exclusion criteria were (i) receiving levofloxacin (because it is the internal standard for determination of linezolid plasma concentration), (ii) receiving other investigational drugs, and (iii) other factors that the researcher considered unsuitable for inclusion.

In the subsequent efficacy and safety analysis, all patients in the PPK analysis were involved, except those who lacked complete clinical information or had specific comorbidities, such as Kawasaki disease, autoimmune diseases, and cancer, which can cause significant abnormalities in blood routine.

Linezolid (Zyvox; Pfizer Inc.) was intravenously infused at a standard dose (10 mg/kg, q8h, i.v., for children under 12 years; 600 mg, q12h, i.v., for children aged 12 years and above). Blood samples were collected using an opportunistic sampling strategy when the steady-state linezolid concentration was achieved (at least 48 h from the start of treatment) (12). Then, plasma was separated by centrifugation for 10 min at 3,000 rpm immediately before it was stored at −80°C.

Precise administration and sampling time were recorded prospectively. In addition, we collected clinical and demographic data, including gender, age, weight (WT), height (HT), aspartate aminotransferase (AST), alanine aminotransferase (ALT), albumin (ALB), total bilirubin (TBIL), direct bilirubin (DBIL), serum creatinine concentration (SCR), blood urea nitrogen concentration (BUN), white blood cell (WBC), platelets (PLT), neutrophil percentage (NEUT%), C-reactive protein (CRP), fever, duration of medication, duration of hospitalization, discharge diagnosis, and discharge disposition. Estimated glomerular filtration rate (eGFR) was calculated using the Schwartz formula. The time points for data collecting were baseline (−3–1 day), initial stage (24–72 h), and termination of treatment (the beginning of linezolid treatment was set as 0 h).

### Analytical method of linezolid

Linezolid concentrations were measured using high-performance liquid chromatography method (LC-2030C NT; Shimadzu Inc.) with ultraviolet detection. The internal standard was levofloxacin. The analytical column was a C18 column (250 × 4.6 mm, 5 µm). The detection wavelength was 254 nm. The mobile phase consisted of 0.05 mol/L sodium dihydrogen phosphate (pH 4.52, 1% phosphoric acid) and acetonitrile in a ratio of 78:22. The flow rate was 1.0 mL/min. The lower limit of quantitation was 0.25 µg/mL. The linear range of the standard curve was 0.25–50 μg/mL.

### Population pharmacokinetic modeling of linezolid

Non-linear mixed-effects modeling program NONMEM v7.4 (Icon Development Solutions, San Antonio, TX, USA) was used for PPK analysis, and the first-order conditional estimation method with interaction was applied to estimate pharmacokinetic parameters and their variability.

One- and two-compartment models with first-order elimination were attempted on the basis of previously reported PPK models (13, 14). The initial model was selected based on the precision and plausibility of parameter estimation, as well as the improvement in the objective function value (OFV).

Interindividual variability of the PK parameters was estimated using an exponential model, expressed as follows:


(1) $$\theta_{i}\;=\;\theta_{mean}\;\times \;e^{\eta i},$$

where *θ*_*i*_ represents the individual PK parameter value, *θ*_mean_ is the typical population value representing a fixed effect in the population, and *ηi* denotes the variability between individuals following a normal distribution.

Residual variability was estimated using an exponential model, addition model, and mixed model, respectively. The optimal residual model was determined by analyzing the OFV.

Covariate analysis employed a stepwise forward inclusion and backward elimination method. The model considered the following covariates: gender, age, WT, HT, AST, ALT, ALB, TBIL, DBIL, SCR, eGFR, and BUN. During covariate model construction, if the OFV decreased by more than 3.84 (*P* < 0.05), indicating significant improvement in model fit, the covariate was retained; otherwise, it was removed. After adding all covariates with statistical significance to the linezolid model, backward elimination was performed. Each covariate was sequentially removed, and if the OFV increased by more than 6.63 (*P* < 0.01), the variable was retained in the final model.

### Model validation

The goodness-of-fit was evaluated using diagnostic scatter plots, which included observed concentrations (DV) versus population-predicted concentrations (PRED), DV versus individual-predicted concentrations (IPRED), conditional weighted residuals (CWRES) versus time since the last dose (TSLD), and CWRES versus PRED plots. The stability and performance of the final model were assessed using nonparametric bootstrap resampling (*n* = 1,000) with replacement. The predictability of the final model was evaluated using the normalized prediction distribution error (NPDE) test. One thousand simulations were conducted based on the final model parameters. NPDE values were expected to follow a normal distribution *N* (0,1). The NPDE results were graphically summarized using the R program (v 4.2.2), including quantile–quantile (Q-Q) plots of NPDE and NPDE histograms. In addition, a visual predictive check (VPC) was performed using R/Xpose, and the output came from PsN in the Pirana environment.

### Simulation and dosing regimen optimization

Monte Carlo dose simulations were conducted 1,000 times to optimize various dosage regimens. We chose AUC_0-24h_/minimum inhibitory concentration (MIC) ≥80 (a ratio of the area under the curve over 24 h to the minimum inhibitory concentration is equal to or greater than 80) as the PD target (13) and *C*_min_ = 7 µg/mL (trough concentration is equal to 7 µg/mL) as the safety threshold (15). Linezolid is used to treat infectious diseases caused by Gram-positive bacteria, such as *Staphylococcus aureus*, *Streptococcus pneumoniae*, and vancomycin-resistant *Enterococcus* (3, 5). According to the Clinical and Laboratory Standards Institute (CLSI) standards, MIC values ranging from 0.06 to 4 µg/mL can encompass all aforementioned pathogens (16). Therefore, we conducted simulations within this range.

The simulation was conducted on two groups as follows: children under 12 years who use linezolid based on mg/kg data, and children aged 12 years and above who use a constant dosage with varying weights. If the currently used dosage is insufficient, the dosing regimen will be adjusted by increasing the dose or frequency. The calculation of the AUC_0-24h_ was as follows:


(2) $$AUC_{0-24h}=\frac{Dose_{0-24h}}{CL},$$

where AUC_0-24h_ is the area under the curve in 0–24 h after administration of linezolid, Dose_0-24h_ is the daily dose of linezolid, and CL is the clearance of linezolid.

### Efficacy and safety of standard dose of linezolid

The primary outcome was the probability of target (AUC_0-24h_/MIC ≥ 80) attainment (PTA). Taking into account the low positivity rate of bacterial cultures in children and the lack of drug sensitivity results, we referred to the MIC values from published articles and standards. As reported by CLSI, MIC breakpoints of *Staphylococcus aureus*, *Streptococcus pneumoniae*, and *Enterococci* are 4, 2, and 2 µg/mL, respectively (16). Previous studies revealed that for linezolid, although the upper limit of the MIC range is 4 µg/mL, nearly all pathogens are equal to or less than 2 µg/mL (16–18). Consequently, we chose MIC = 2 µg/mL as the reference value for calculating the PD target. PD target (AUC_0-24h_/MIC ≥ 80) attainment was evaluated using the individual empirical Bayesian estimates method with the PPK model by the NONMEM software.

Secondary outcomes included the following: (i) discharge disposition, (ii) clinical responses (e.g., fever, WBC, NEUT%, CRP), (iii) the probability of surpassing the safety threshold (*C*_min_ = 7 µg/mL), and (iv) incidence of AEs (e.g., vomiting, diarrhea, abdominal pain, confusion, anemic, thrombocytopenia, lactatemia, and serotoninergic syndrome). Discharge disposition was delineated into the following three categories: cured, improved, or failure. Cured meant the signs and symptoms of infection were completely resolved. Improvement was characterized by significant improvement in the signs and symptoms of infection. Failure, on the other hand, was defined as either non-response to treatment or exacerbation of the condition (18). In addition, we evaluated clinical responses, which included fever, WBC, NEUT%, and CRP, at the beginning (48–72 h after the initial treatment) and the end of linezolid treatment. Improved clinical responses were defined as the presence of fever or elevated laboratory parameters before treatment, followed by a decrease in peak temperature or parameter values after treatment. Failure was defined as an increase in peak temperature or parameter values after treatment. Normal meant body temperature and laboratory parameters were within the reference range during the administration (19). Safety threshold (*C*_min_ = 7 µg/mL) was also evaluated using the individual empirical Bayesian estimates method with the PPK model by the NONMEM software. The World Health Organization–Uppsala Monitoring Center system for standardized case causality assessment was used to evaluate suspected AEs (20). The definitions of anemic, lactatemia, and serotoninergic syndrome reference UpToDate (21–23). Thrombocytopenia was evaluated at the end of the treatment, and it was defined as a reduction of platelet count to <112.5 × 10^9^ /L (75% of the lower limit of normal) for patients whose baseline platelet counts were in the normal range. For patients with baseline platelet counts below the lower limit of normality, thrombocytopenia was defined as a decrease in platelet count of ≥25% from the baseline value (18, 24).

### Statistical analysis

Data were expressed as the median (5th–95th). Proportions were compared by the χ^2^ or Fisher’s exact tests. NPDE was tested by Wilcoxon signed rank test, Fisher’s exact test, Shapiro–Wilk normality test, and multiple tests corrected by Bonferroni correction. Statistical significance was defined by a two-sided *P* value of 0.05. The statistical analyses were performed using IBM SPSS v23.0 (IBM Corp., Armonk, NY, USA) and R program (v 4.2.2).

## RESULTS

### Study population

A total of 80 patients were included in the PPK study. The median (5th–95th) age and weight of patients were 3.3 (0.1–12.6) years and 12.5 (5.3–45.9) kg, respectively. Detailed demographic characteristics of patients in the PPK study are presented in Table 1.

**TABLE 1:** Demographic and clinical characteristics of patients in the PPK study^*a*^

Characteristic	No.	Median (5th–95th)
Patients	80	
Samples	157	
Gender		
Male	48	
Female	32	
Samples for per patient		2.0 (1.1–2.0)
Age (year)		3.3 (0.1–12.6)
WT (kg)		12.5 (5.3–45.9)
HT (cm)		99.5 (55.1–159.8)
Linezolid dose (mg/kg/dose)	73	10.0 (9.4–10.5)
Linezolid dose (mg/day)	7	600.0 (600.0–600.0)
AST (U/L)		26.0 (12-117)
ALT (U/L)		15.3 (5.4–170.0)
ALB (g/L)		34.0 (25.5–43.5)
TBIL (μmol/L)		6.5 (2.4–37.1)
DBIL (μmol/L)		1.7 (0.5–15.0)
SCR (μmol/L)		24.0 (9.0–48.0)
eGFR (mL/min/1.73 m^2^)		190.1 (97.0–350.2)
BUN (mmol/L)		2.7 (1.0–5.9)

^*a*^ WT, weight; HT, height; AST, aspartate aminotransferase concentration; ALT, alanine aminotransferase concentration; ALB, albumin; TBIL, total bilirubin; DBIL, direct bilirubin; SCR, serum creatinine concentration; eGFR, estimated glomerular filtration rate; BUN, blood urea nitrogen.

A total of 67 patients were included in the efficacy and safety analysis. The most common diseases were pulmonary infections (32, 47.8%), central nervous system infections (21, 31.3%), and sepsis (21, 31.3%). Other details are displayed in Table 2.

**TABLE 2:** Demographic and clinical characteristics of patients in the efficacy and safety analysis^*a*^

Characteristic	No. (%)	Median (5th–95th)
Patients	67	
Gender		
Male	42 (62.7%)	
Female	25 (37.3%)	
Age (year)		3.3 (0.1–12.6)
WT (kg)		12.5 (5.3–48.1)
HT (cm)		99.5 (55.1–157.1)
Linezolid dose (mg/kg)	61 (91.0%)	10.0 (9.5–10.5)
Linezolid dose (mg)	6 (9.0%)	600.0 (600.0–600.0)
Duration of medication (day)		9.0 (2.0–30.0)
Duration of hospitalization (day)		15.0 (3.0–43.6)
WBC		12.2 (3.3–23.2)
NEUT%		66.5 (18.7–89.8)
CRP		51.3 (1.6–244.4)
PLT		308.0 (51.8–587.4)
Types of infectious diseases		
Pulmonary infections	32 (47.8%)	
Central nervous system infections	21 (31.3%)	
Sepsis	21 (31.3%)	
Osteomyelitis	9 (13.4%)	
Skin and soft tissue infection	8 (11.9%)	
Lymphnoditis	5 (7.5%)	
Urinary infection	4 (6.0%)	
Tuberculosis	2 (3.0%)	
Acute gangrenous appendicitis	1 (1.5%)	
Infectious endocarditis	1 (1.5%)	
Craniocerebral injury	1 (1.5%)	
Pathogens^*b*^		
*Staphylococcus aureus*	13 (19.4%)	
*Streptococcus pneumoniae*	5 (7.5%)	
Methicillin-resistant *Staphylococcus aureus*	4 (6.0%)	
*Enterococcus faecium*	3 (4.5%)	
*Enterococcus faecalis*	1 (1.5%)	
*Staphylococcus epidermidis*	1 (1.5%)	
*Saprophytic Staphylococcus*	1 (1.5%)	
*Viridans streptococci*	1 (1.5%)	
*Streptococcus mitis*	1 (1.5%)	

^*a*^ WT, weight; HT, height.

^*b*^ The pathogens here are the results of bacterial cultures.

### Model building

**Fig 1:**
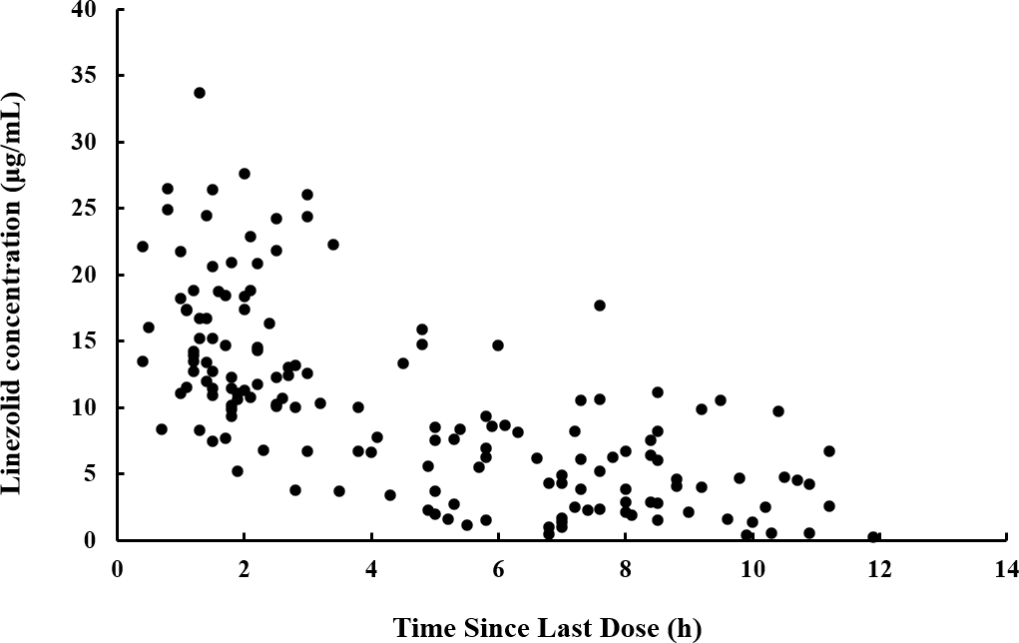
Linezolid concentrations versus time since last dose.

A total of 157 plasma samples, ranging from 0.25 to 33.67 µg/mL, were utilized in the PPK modeling. The linezolid concentration versus time since last dose profile is shown in Fig. 1. A one-compartment model with first-order elimination was most suitable for describing the PK characteristics of linezolid. The model was parameterized in terms of clearance (CL) and volume of distribution (V), and exponential models were used for both interindividual variation and residual variation.

In the covariate analysis, we considered 12 covariables as follows: gender, age, WT, HT, AST, ALT, ALB, TB, DB, SCR, eGFR, and BUN. In the forward selection process, WT and eGFR caused a significant drop in OFV of 77.376 and 21.119 points, respectively. None of the other evaluated covariates had any significant influence on the optimization of the model. In the backward elimination procedure, WT and eGFR were retained in the final model as significant covariates. The estimated parameters of the final model of linezolid in children with bacterial infections are presented in Table 3.

**TABLE 3:** Parameter estimates of the final PPK model and bootstrap results^*a*^

Parameter	Final model		Bootstrap analysis	
	Estimate	RSE (%)	Median estimate	5th–95th
CL (L/h)				
CL = θ1 × (WT/12.5)^θ3^ × (eGFR/190.1)^θ4^				
θ1	1.91	5.1	1.88	45.095
θ3	0.696	10.6	0.701	0.348–1.09
θ4	0.291	36.1	0.309	0.01–0.983
*V* (L)				
*V* = θ2				
θ2	10.5	11.8	10.5	4–194.2
Inter-individual variability (%)				
CL	33.02	37.7	33.02	0.71–168.39
*V*	82.28	33.8	81.02	0.55–132.16
Residual variability (%)	26.23	69.6	28.54	13.52–106.25

^*a*^ RSE relative standard error; CL, clearance; V, Volume of distribution; WT, weight; eGFR, estimated glomerular filtration rate. In our population, 12.5 kg and 190.1 mL/min/1.73 m^2^ are the median WT and eGFR values, respectively.

### Model evaluation

**Fig 2:**
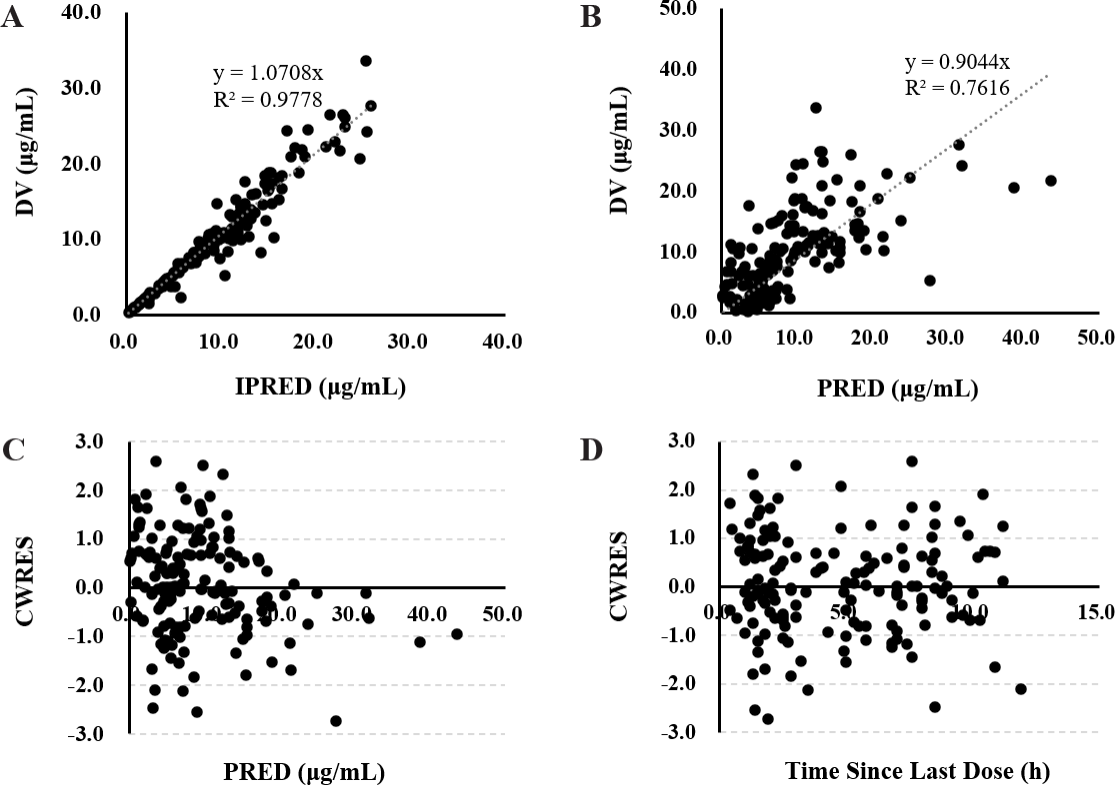
Goodness-of-fit plot for the final model. (**A**) Observed concentrations (DV) versus individual prediction (IPRED); (**B**) DV versus population prediction (PRED); (**C**) conditional weighted residuals (CWRES) versus PRED; (**D**) CWRES versus time since last dose.

**Fig 3:**
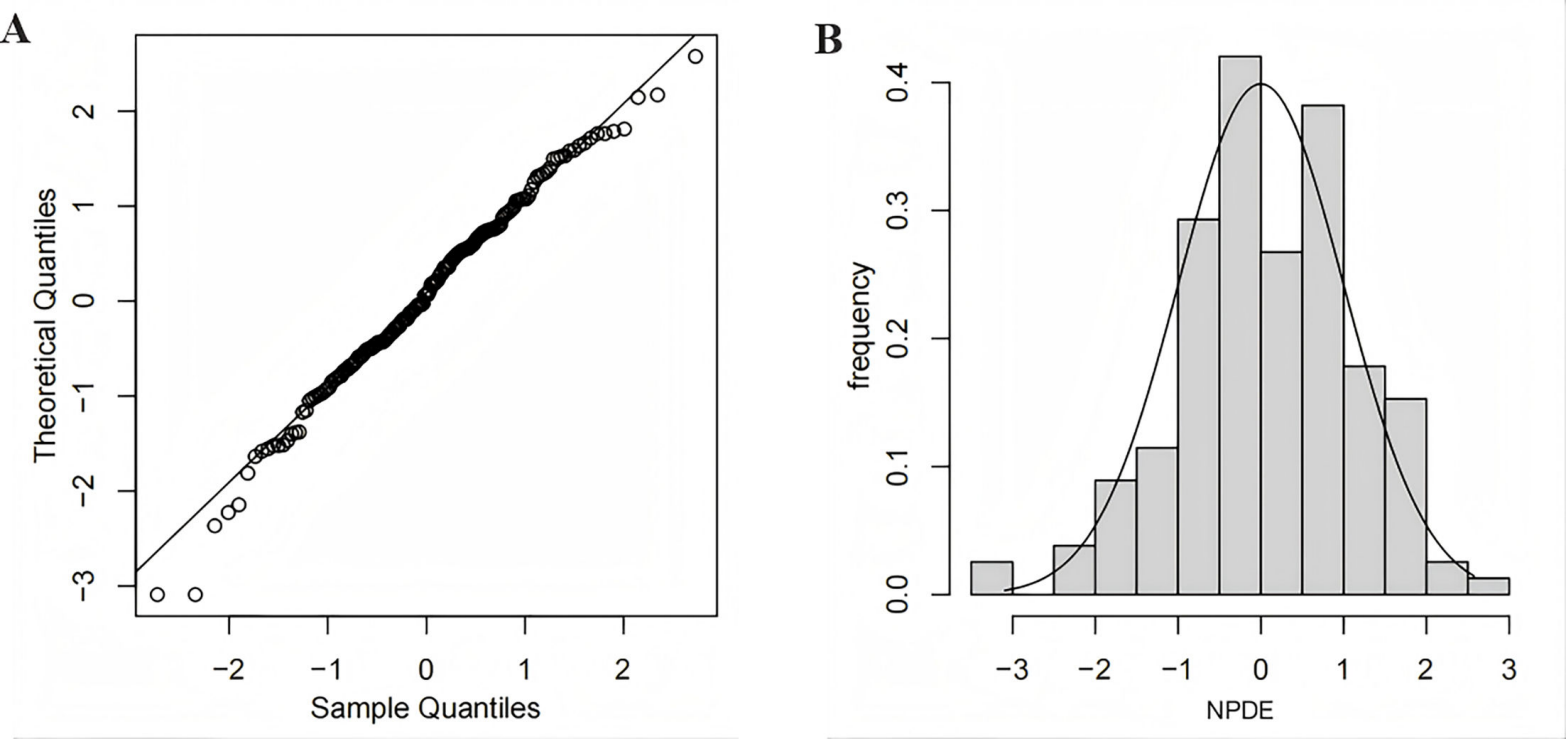
Normalized prediction distribution errors (NPDE) of the final population pharmacokinetic model. (**A**) Q-Q plot of NPDE vs theoretical *N* (0,1) distribution. (**B**) Histogram of the distribution of NPDE.

**Fig 4:**
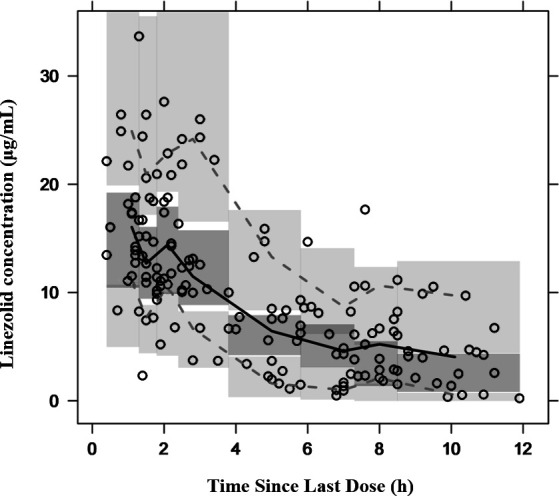
Visual predictive check (VPC) of the final model. The spots represent the observed linezolid concentrations. Solid and dashed lines represent the 50th percentile and the 5th and 95th percentiles, respectively, of the observed concentrations. The three shaded areas represent the 95% confidence intervals of the 5th, 50th, and 95th percentiles of the simulated concentrations.

Model diagnostics showed that the final model of linezolid had an acceptable goodness of fit. Figure 2A and B shows no bias in predicting concentration. Figure 2C and D shows that there is no trend between PRED and CWRES or between time and CWRES. The results of NPDE are shown in Fig. 3. The mean value of NPDE was 0.0652 (Wilcoxon signed rank test *P* = 0.285), and the variance was 1.08 (Fisher variance test *P* = 0.445), Shapiro–Wilks test *P* = 0.336, Global adjusted *P* = 0.855, which indicated that the model followed a normal distribution *N* (0,1), that is, the model was consistent with individual data. In addition, as shown in Table 3, the median values of the parameters estimated by a bootstrap method were consistent with the corresponding values of the final model, indicating that the final model was stable and that the parameters estimated by the PPK model could be reconfirmed. The VPC result of the final model (Fig. 4) showed that the 5th, 50th, and 95th percentiles of the observed concentration were within 95% confidence interval of the predicted concentration, which proved the accuracy and adaptability of the model.

### Dosing regimen evaluation and optimization

**Fig 5:**
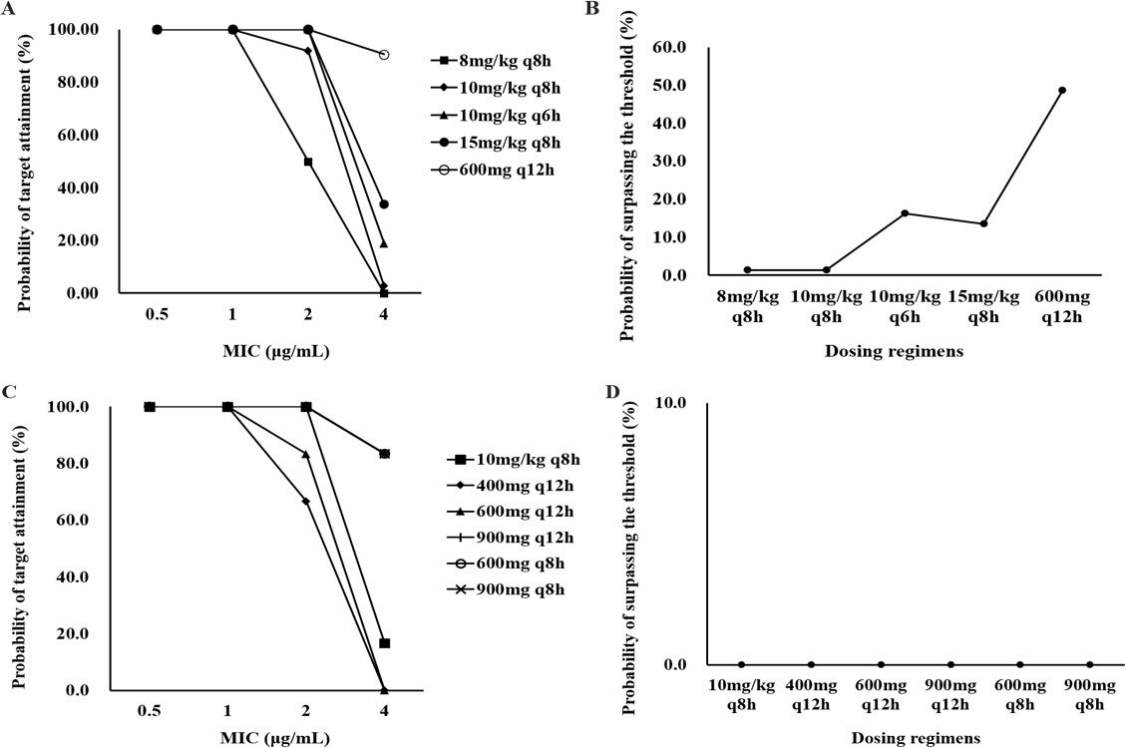
The results of dose simulation and optimization. (**A**) Probability of target (AUC_0 –24_/MIC ≥ 80) attainment for various dosing regimens in children under 12 years. (**B**) Probability of surpassing the threshold (*C*_min_ = 7 µg/mL) for various dosing regimens in children under 12 years. (**C**) Probability of target (AUC_0 –24_/MIC ≥ 80) attainment for various dosing regimens in children aged 12 years and above. (**D**) Probability of surpassing the threshold (*C*_min_ = 7 µg/mL) for various dosing regimens in children aged 12 years and above.

Monte Carlo simulations were conducted to simulate different dosing regimens. Figure 5A and C illustrates the relationship between dosing regimens and PTA (AUC_0-24h_/MIC ≥80), across various MIC values ranging from 0.5 to 4 µg/mL. Additionally, Fig. 5B and D presents the probability of surpassing the safety threshold (*C*_min_ = 7 µg/mL) for different dosing regimens.

In children under 12 years, the PTA for standard dosing regimen (10 mg/kg, q8h) was 100.0%, 100.0%, and 91.9% when the MIC was 0.5, 1, and 2 µg/mL, respectively, with a mere 1.4% probability of surpassing the safety threshold. When dealing with pathogens exhibiting an MIC of 4 µg/mL, only the dosage of 600 mg q12h was capable of achieving a satisfactory PTA of 90.5%. Nonetheless, concurrently, the probability of surpassing the safety threshold soared to 48.6%. Higher dosages may thus be imperative, particularly when confronted with pathogens bearing an MIC of 4 µg/mL. Higher dosages also carried a significantly increased risk of surpassing the safety threshold, so it will be essential for optimal antimicrobial therapy to strike a balance between efficacy and safety.

In children aged 12 years and above, the PTA for standard dosage (600 mg, q12h) was 100.0%, 100.0%, and 83.3% at MIC levels of 0.5, 1, and 2 µg/mL, respectively. The probability of surpassing the safety threshold was 0.0%. However, the PTA was 0.0% at an MIC of 4 µg/mL. Increasing the dosage to 900 mg q12h or 600 mg q8h could achieve PTAs of 83.3% and 83.3% at an MIC of 4 µg/mL. Meanwhile, the probability of surpassing the safety threshold was 0.0%.

### Efficacy and safety study

A total of 67 patients were convened for efficacy and safety analysis, the PTA was determined to be 76.1% (51/67, AUC_0-24h_/MIC ≥80, MIC = 2 µg/mL), and the probability of surpassing the safety threshold was 14.9% (10/67, *C*_min_ = 7 µg/mL).

Among the patients, 6 (9.0%) were cured at the time of discharge, and 58 (86.6%) showed significant improvement, with a PTA of 100.0% and 74.1%, respectively (Table 4). The AUC/MIC between any two groups was not found to be statistically significant. Throughout the treatment course, there were also noticeable enhancements in clinical responses. At the onset of treatment (48–72 h after the initial treatment), failure rates for fever, WBC, NEUT %, and CRP were 10.4%, 13.4%, 19.4%, and 7.5%, respectively. By the end of the treatment, these rates were 1.5%, 20.9%, 9.0%, and 3.0%, respectively (Table 5). These findings underscore the efficacy of the linezolid standard dose (10 mg/kg, q8h, i.v., for children under 12 years; 600 mg, q12h, i.v., for children aged 12 years and above).

**TABLE 4:** Prognosis and PTA of children with linezolid therapy^*a*^

Prognosis	*n*/*N* (%)	AUC/MIC (median, 5th–95th)^*b*^	PTA (*n*/*N*, %)
Cured	6/67 (9.0%)	105.6 (80.4–163.5)	6/6 (100.0%)
Improved	58/67 (86.6%)	133.5 (67.8–134.0)	43/58 (74.1%)
Failure	3/67 (4.5%)	103.7 (55.8–187.3)	2/3 (66.7%)

^*a*^ PTA, probability of target (AUC_0-24h_/MIC ≥ 80, MIC = 2 μg/mL) attainment; Cured, complete resolution in the signs and symptoms; Improved, significant improvement in the signs and symptoms of the infection; Failure, either non-response to treatment or exacerbation of the condition.

^*b*^ The differences between any two groups were not statistically significant when MIC = 2 μg/mL.

**TABLE 5:** Clinical response to linezolid therapy^*a*^

Items	Linezolid treatment after 48–72 h			Linezolid treatment completed		
	Improvement *n/N* (%)	Failure *n*/*N* (%)	Normal *n*/*N* (%)	Improvement *n*/*N* (%)	Failure *n*/*N* (%)	Normal *n*/*N* (%)
Fever	52/67 (77.6%)	7/67 (10.4%)	8/67 (11.9%)	59/67 (88.1%)	1/67 (1.5%)	7/67 (10.4%)
WBC	33/67 (49.3%)	8/67 (11.9%)	26/67 (38.8%)	35/67 (52.2%)	6/67 (9.0%)	26/67 (38.8%)
NEUT%	30/67 (44.8%)	10/67 (14.9%)	27/67 (40.3%)	32/67 (47.8%)	4/67 (6.0%)	31/67 (46.3%)
CRP	50/67 (74.6%)	5/67 (7.5%)	12/67 (17.9%)	51/67 (76.1%)	2/67 (3.0%)	14/67 (20.9%)

^*a*^ WBC, white blood cell; NEUT%, neutrophil percentage; CRP, C-reactive protein; Improvement, the presence of fever or elevated laboratory parameters before treatment, followed by a decrease in peak temperature or parameter values after treatment; Failure, the increase in peak temperature or parameter values after treatment; Normal, body temperature and laboratory parameters within the reference range during the treatment.

A total of 15 (15/67, 22.4%) patients developed one or more “possible” AEs, and the most common AEs were anemia (7/67, 10.4%), vomiting (5/67, 7.5%), and diarrhea (4/67, 6.0%) (Table 6). Abdominal pain, confusion, lactatemia, and serotoninergic syndrome were not observed. No children experienced early discontinuation of linezolid due to AEs. Considering these findings, it can be concluded that standard linezolid dosage demonstrates safety in the treatment of bacterial infections in children.

**TABLE 6:** Analysis of adverse events in children after linezolid therapy

Adverse events	*n*/*N* (%)
Anemic	7/67 (10.4%)
Vomit	5/67 (7.5%)
Diarrhea	4/67 (6.0%)
Thrombocytopenia	1/67 (1.5%)

## DISCUSSION

We established the PPK model of linezolid in children with bacterial infections. The results of PPK analysis indicated that a one-compartment model with first-order elimination, along with WT and eGFR as significant covariates for CL, was optimal for PK data of linezolid. The range of weight-normalized estimated CL was 0.13–0.171 L/h/kg and *V* was 0.42–0.92 L/kg, and the values of our study were 0.15 ± 0.06 L/h/kg and 0.77 ± 0.50 L/kg, which are consistent with the findings of a prior PK/PPK study of linezolid in pediatric patients (13, 25, 26).

**Fig 6:**
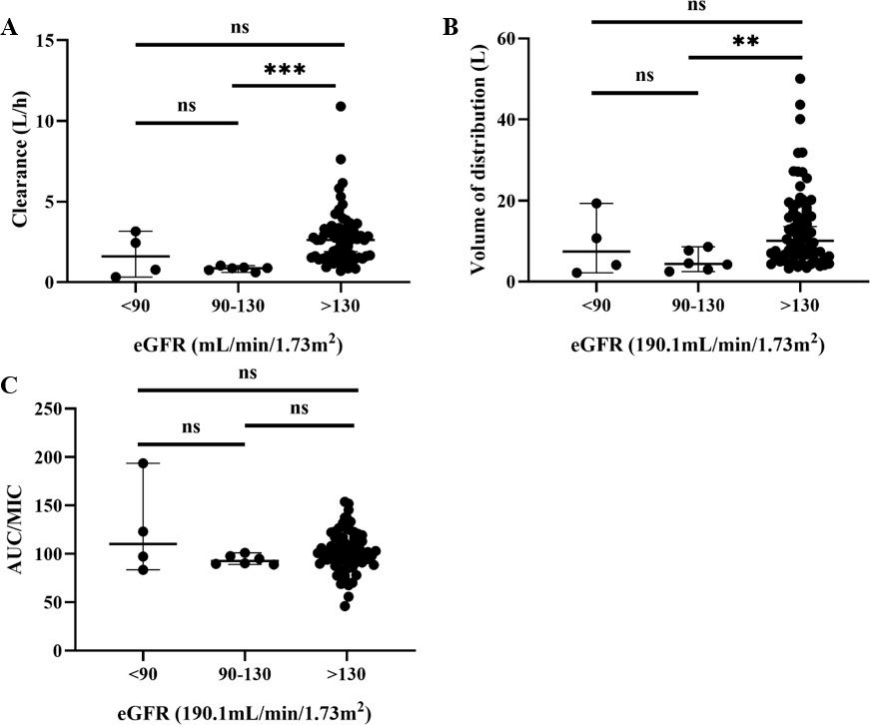
The differences in PK characteristics between population with different renal function. (**A**) Clearance versus estimated glomerular filtration rate (eGFR). (**B**) Volume of distribution versus eGFR. (**C**) AUC/MIC (MIC = 2 µg/mL) versus eGFR. **P < 0.01, ***P < 0.001.

Our covariate analysis has also incorporated WT into the model, as reported in previous studies, especially in the PPK of children (13, 14, 25, 27). Another significant covariate in our model was eGFR. In our study, the median eGFR was 190.1 mL/min/1.73 m^2^, which means that most of the children in this study were in a state of augmented renal clearance (ARC) (28). We compared PK parameters at different renal functions and found that CL and *V* were significantly higher in patients with ARC (eGFR >130 mL/min/1.73 m^2^) than in patients with normal renal function (90 ≤ eGFR ≤ 130 mL/min/1.73 m^2^). However, no statistically significant difference in AUC/MIC was observed between the two groups (Fig. 6). In summary, ARC does affect the metabolism of linezolid in children, but has no effect on PTA. Children hospitalized with infectious diseases often experience ARC, which can increase drug clearance (29, 30). Despite the initial study of linezolid PK in patients with renal dysfunction suggesting that dose adjustment was not necessary because of the similar concentration observed compared to those of healthy volunteers (31), approximately 30% of the dose was excreted as unchanged drug in urine at steady state (Zyvox Product label). Consequently, several PPK studies on linezolid have also incorporated renal function indices into the model, particularly eGFR (13, 32–35).

Previous studies have proposed the PD target for linezolid as a ratio of the area under the curve over 24 h to the minimum inhibitory concentration (AUC_0-24h_/MIC) between 80 and 120 (13, 14, 36–38). Especially, AUC_0-24h_/MIC ≥80 would be more appropriate for children (13, 14). In addition, C_min_ >7 µg/mL was significantly correlated with the occurrence of AEs (15, 39). Based on the aforementioned reasons, we chose AUC_0-24h_/MIC ≥80 as the PD target and *C*_min_ = 7 µg/mL as the safety threshold.

The efficacy and safety analysis, which combined the PD target based on the PPK model and real-world data, showed that patients using standard dose (10 mg/kg, q8h, i.v., for children under 12 years; 600 mg, q12h, i.v., for children aged 12 years and above) reached the PTA of 76.1% (AUC_0-24h_/MIC ≥80, MIC = 2 µg/mL), and 95.5% of the patients got a successful clinical treatment. In addition, 14.9% (10/67) of the patients surpassed the safety threshold (*C*_min_ = 7 µg/mL), 22.4% (15/67) of the patients developed possible AEs, and no patient experienced early discontinuation of linezolid due to AEs. Of the 10 patients with *C*_min_ >7 µg/mL, 5 patients (5/10, 50%) experienced possible AEs, and the most common AEs were vomiting (2/10) and anemia (2/10). A previous meta-analysis including 758 children has reported that the overall proportion of clinical improvement was 88.80% of the patients (95% CI = 81.31%–93.52%), and the incidence of AEs was 8.91% (95% CI = 1.64%–36.52%), which is consistent with our study (40). Higher rates of incidences of AEs will be observed in patients with longer treatment (especially for tuberculosis) and higher dosage (41–43). Based on the results from both theoretical and practical analysis, the standard linezolid dosage is effective and safe for treating children with bacterial infections.

The simulation results indicated that for all children, the standard linezolid dosage can reach the well efficacy and safety target when MIC is equal to and less than 2 µg/mL. However, when children were infected by a pathogen with an MIC of 4 µg/mL, 600 mg q12h (for children aged under 12 years) and 900 mg q12h or 600 mg q8h (for children aged 12 years and above) were capable of achieving a satisfactory PTA. Concurrently, the rates of surpassing safety threshold were 48.6% and 0.0%. Based on the outcomes above, for bacteria with an MIC ≤ 2 µg/mL, the standard dosage is feasible; for bacteria with an MIC > 2 µg/mL, considering both efficacy and safety, changing antibiotics is recommended for children under 12 years old, while for individuals aged 12 years and above, a dose regimen of 900 mg q12h or 600 mg q8h can be adopted. Previous studies have also reported that the PTA of linezolid standard dosage was above 95% when MIC ≤ 1 µg/mL. However, in the presence of pathogens exhibiting an MIC of 2 µg/mL or greater, a higher dose or alternative antimicrobial would be required (13, 14). Different from previous studies, our study reported that the standard dosage was able to reach an adequate drug exposure when MIC was 2 µg/mL, and we recommend no change at this stage. The reasons are as follows: to evaluate the results of dose simulation, we conducted the efficacy and safety analysis in real-world data, and the results (95.5% of patients got a successful clinical treatment; 22.4% of patients developed possible AEs, and no patient experienced early discontinuation of linezolid due to AEs) showed that standard linezolid dosage was effective and safe enough when MIC is 2 µg/mL. In addition, our PTA was similar to that of the previous results, and we ran the safety simulation, which shows that increasing the dose slightly increases the PTA, but the safety risk increases substantially.

This study successfully developed a PPK model for linezolid in children and subsequently evaluated the simulation outcomes in clinical practice. Nevertheless, due to the limited sample size, our model was not validated externally. Furthermore, future research should be conducted with larger sample sizes in clinical practice, particularly focusing on children aged 12 years and above who are infected with pathogens having an MIC greater than 2 µg/mL, to provide more definitive results.
